# Advancements in Subchondral Bone Biomechanics: Insights from Computed Tomography and Micro-Computed Tomography Imaging in Equine Models

**DOI:** 10.1007/s11914-024-00886-y

**Published:** 2024-09-14

**Authors:** Fatemeh Malekipour, R. Chris Whitton, Peter Vee-Sin Lee

**Affiliations:** 1 Department of Biomedical Engineering, University of Melbourne, Parkville, VIC 3010 Australia; 2 Equine Centre, Department of Veterinary Clinical Sciences, University of Melbourne, Werribee, VIC 3030 Australia

**Keywords:** Subchondral bone (SCB), Subchondral bone microstructure, Osteoarthritis; subchondral bone in stress fracture, Micro-computed tomography imaging, Computed tomography imaging, Equine joint biomechanics

## Abstract

**Purpose of Review:**

This review synthesizes recent advancements in understanding subchondral bone (SCB) biomechanics using computed tomography (CT) and micro-computed tomography (micro-CT) imaging in large animal models, particularly horses.

**Recent Findings:**

Recent studies highlight the complexity of SCB biomechanics, revealing variability in density, microstructure, and biomechanical properties across the depth of SCB from the joint surface, as well as at different joint locations. Early SCB abnormalities have been identified as predictive markers for both osteoarthritis (OA) and stress fractures. The development of standing CT systems has improved the practicality and accuracy of live animal imaging, aiding early diagnosis of SCB pathologies.

**Summary:**

While imaging advancements have enhanced our understanding of SCB, further research is required to elucidate the underlying mechanisms of joint disease and articular surface failure. Combining imaging with mechanical testing, computational modelling, and artificial intelligence (AI) promises earlier detection and better management of joint disease. Future research should refine these modalities and integrate them into clinical practice to enhance joint health outcomes in veterinary and human medicine.

## Introduction

Subchondral bone (SCB) is located adjacent to the articular surface within diarthrodial joints. While uncalcified cartilage offers a cushioning, avascular layer at the bone ends, tissue stiffness progressively increases with greater depth transitioning into the calcified cartilage layer, then the subchondral plate and the underlying trabecular bone [1, 2]. This specialised microstructure of the SCB, including both the subchondral plate and its underlying trabecular bone, provides a foundation for the articular cartilage, which is crucial for maintaining joint integrity, congruity and function during locomotion [3, 4].

SCB exhibits a complex structure–function relationship. For example, mechanical properties of SCB in athletic horses are not explained by its microstructure and mineral content alone and are heavily influenced by the presence of microdamage [2, 5]. More importantly, the relationship between mechanical and microstructural properties varies across the depth of the SCB from the articular surface. For example, within the 10 mm superficial SCB, the relationship may change for every 2 to 3 mm of depth [2, 6, 7, 8]. These unique properties likely allow mechanical load to distribute favourably within the entire joint, thus playing a vital role in maintaining joint integrity and influencing the onset and progression of joint disorders, such as osteoarthritis (OA) and stress fractures [9, 10]. Therefore, alterations in SCB structure can impact cartilage-bone functionality affecting overall joint health [11, 12] and early detection of changes in the structure of the SCB can help with a better management of musculoskeletal disorders.

Historically, the complexity of SCB microstructure and bone mineral density (BMD) was primarily assessed using two-dimensional radiographic and histological techniques, which provided limited sensitivity [13]. The advent of computed tomography (CT) and micro-computed tomography (micro-CT) in the late twentieth century marked a significant enhancement, enabling non-destructive three-dimensional visualization of this complex architecture [14, 15]. In addition to macroscale CT imaging, micro-CT imaging has significantly enhanced the study of the microstructure of SCB. Most importantly, micro-CT enables both pre- and post-mechanical test imaging, a capability not available in earlier studies that relied on destructive histological analyses [2, 16]. This advancement allows for a more detailed and non-destructive examination of SCB, preserving the integrity of samples for further analysis.

The use of large animal models, such as pigs, sheep, dogs, and horses, has proven invaluable in studying SCB. Large animals are critical for modelling the complex disease mechanisms that affect functional joints, especially in translational research [17, 18, 19, 20]. Each species, including dogs, minipigs, sheep, goats, and horses, has unique anatomical and functional characteristics that make them valuable for specific research questions. For instance, these animals share similarities with humans in terms of joint anatomy, biomechanical function, cartilage and SCB morphology, and responses to injury. These similarities make large animals more clinically relevant for studying SCB and articular cartilage disorders compared to smaller animal models [17, 21].

The objective of this review is to synthesise the literature published over the past three years focusing on SCB microstructure in large animals, with a particular emphasis on horses. This review utilizes findings from both micro-CT and CT imaging. It is important to note that this review is not intended to be systematic, but rather to provide a broad overview of recent advancements and insights in this field. The review focuses on equine models due to their high prevalence of musculoskeletal injuries [22, 23]. More importantly, horses provide a naturally occurring model of OA that resembles human disease on both a macroscopic and a histological level [22, 24]. Joint disease in racehorses, caused by repeated intensive exercise, provides a model to study repetitive stress-induced changes in cartilage and SCB. Additionally, horses are excellent for studying biomechanics due to their size and athletic nature [25, 26], including research into gait analysis, stress on joints during motion, and the impact of various training regimens.

### Role of SCB in Stress Fracture and OA

Stress fractures are a common injury affecting the integrity of the SCB. In athletic horses, these fractures typically occur due to excessive repetition of normal loads of galloping, on either well adapted bone, or in some instances poorly adapted bone where the SCB has not been conditioned to the applied loading [13, 27]. Particularly, in racehorses, palmar/plantar osteochondral disease (POD) and third metacarpal/tarsal condylar fractures are considered fatigue injuries of SCB and calcified cartilage [28]. The excessive repetition of load can lead to micro-damage that accumulates over time, progressing to stress fractures or focal SCB injuries if the rate of accumulation of damage exceeds the rate of damage repair by the bone remodelling process [22, 29]. Many equine stress fractures arise from articular surfaces and pre-existing pathology is often observed in the SCB at sites where the fractures have propagated from. Early and accurate detection of these lesions in the joint will help to understand the mechanisms of injury as well as to assess injury risk.

Another joint disease that is directly associated with SCB integrity is OA, a degenerative joint disease characterized by the breakdown of cartilage and changes in the SCB, leading to pain and loss of mobility. SCB alterations play a critical role in both the initiation and progression of OA [30, 31]. Changes in the SCB, such as increased turnover, altered mineralization, and microarchitectural deterioration, often precede and predict articular cartilage degradation. Similarly, in early stages of POD in racehorses, SCB abnormalities occur whilst the articular cartilage, remains grossly intact (Turlo et al., 2022). These early changes in the SCB structure and microstructure, highlight the vital role of identifying and measuring these changes both in vivo and in vitro for clinical and research purposes.

### CT imaging in SCB Stress Fractures and OA in Equine Athletes

CT is an imaging technique that captures cross-sectional images using X-ray photon attenuation. As a transformative tool in SCB research and veterinary diagnostics, CT imaging enables the differentiation of bone and soft tissue in three-dimensions, as well as identifying subtle SCB structural changes and BMD variations, including lesions and complex fractures not visible on radiographs [32, 33, 34, 35]. For example, POD lesions in the fetlock joints of racehorses, manifest as areas of reduced density on radiographs and CT scans, surrounded by trabecular bone with increased density [36, 37, 38]. Additionally, CT imaging offers a thorough assessment of fracture characteristics and associated SCB pathology, aiding in preoperative evaluation and prognostication [36]. Table 1 provides a comparison of the different CT methods used in the study of SCB biomechanics. It outlines typical use-cases, advantages, limitations, and relevant references for each method.

**Table 1:** Overview of various CT Methods in Subchondral Bone Biomechanics

CT Method	Typical Use-Cases	Pros	Cons	
Conventional CT	Differentiation of bone and soft tissue in 3D, identification of SCB structural changes and BMD variations, including SCB injury and fractures	Provides detailed 3D visualization, non-destructive imaging, helpful for early detection and prognostication	Requires general anesthesia for large animals, which carries associated risks and costs	[32, 33, 34, 35, 36, 37, 38]
Standing CT (Helical Fan Beam CT—FBCT)	Scanning large animals without general anesthesia. Used as part of lameness diagnosis in horses	Allows for quick and practical diagnostic imaging in standing animals, without the need for anesthesia	Patient movement can compromise the quality of the scan, though less sensitive to motion artifacts compared to CBCT	[39–41], [45]
Cone-Beam CT (CBCT)	Longitudinal monitoring of SCB changes	Fast acquisition of volumetric data, less radiation exposure	Lower image quality compared to FBCT, especially sensitive to motion artifacts, reduced specificity	[35, 42, 43, 44], [46]
Micro-CT	Detailed visualization of SCB microarchitecture, useful in pre- and post-mechanical test imaging in research settings	High-resolution imaging capable of visualizing bone microarchitecture and microdamage with great detail	Not suitable for live large animal imaging, may not accurately predict SCB mechanical response due to limitations in assessing fine microcracks	[2, 5, 16, ﻿^56^, ^57^, ^58^, ^59^, ^60^, ^61^, ^62^, ^63^]

One of the most significant advancements in CT technology for equine limb imaging is standing CT. This innovation has transformed the practical application of CT in both clinical and research settings by allowing large animals to be scanned without the need for general anaesthesia [39, 40, 41]. Using a helical fan beam CT (FBCT) system, standing CT systems proved effective for diagnosing lameness in horses due to their ease of operation and quick diagnostic capabilities [39]. The system was able to safely scan sedated standing horses from the carpal or tarsal region distally, enabling it to be incorporating into lameness investigations, achieving clinical diagnoses for distal limb lameness.

Most recently, the effectiveness of standing cone-beam CT (CBCT) systems in equine SCB diagnosis and research has been explored. In conventional FBCT, the X-ray source and a curved detector rotate 360 degrees around the subject, taking overlapping images as either the subject moves through the beam or the beam moves over the subject. These images are combined to create a volumetric view. CBCT, however, involves a cone-shaped beam and a flat panel sensor that rotate less than 240 degrees around the subject. FBCT generally produces images with higher quality, including less artifact, lower noise levels, and better contrast detection than CBCT [35, 42, 43, 44]. An important advantage of FBCT is its speed in scanning each cross-section, which is crucial when imaging standing horses, while a slight movement of the horse in a standing CBCT can compromise the quality of the entire scan. Conversely, CBCT is fast in acquisition of volumetric data, e.g. < 1 min of total scan time for the distal limb or caudal neck of a standing, sedated horse [43]. The study found “excellent” satisfaction scores as determined qualitatively by radiologists for bone evaluation in both modalities. Compared to histological measurements, Lin et al. (2023) found that CBCT had slightly higher diagnostic sensitivity (88.5% vs. 84.1%) but lower specificity (61.3% vs. 72.3%) compared to FBCT [45]. Both studies were limited by their focus on cadavers, which are unaffected by motion artifacts which are more problematic in CBCT. Imaging standing Thoroughbred racehorses, a robotic CBCT was used for longitudinal monitoring of SCB adaptation to exercise [46]. Over the first year of training, SCB changes in all four limbs of racehorses at multiple intervals (0, 6 and 12 months) were tracked. Although the exact details of the training regimens were not quantitatively described, the study demonstrated that SCB sclerosis significantly increased over time, and the presence of SCB pathology also escalated in various parts of the metacarpal/metatarsal bone, particularly within the first six months​​.

Given these insights, the integration of advanced CT technologies like standing FBCT and CBCT into diagnostic protocols for equine OA and other SCB pathologies promises to enhance early detection and management. While FBCT offers detailed imaging that may remain superior for certain diagnostic needs, the rapid imaging capabilities, and adequate resolution of CBCT may be suitable for routine screening and monitoring of known conditions. Future research should focus on enhancing the specificity and sensitivity of these imaging modalities, exploring their application in live diagnostic settings, and expanding their use in longitudinal studies to better understand the progression of SCB conditions over time. Despite these advancements, further research is required to enable identification of horses at high risk of SCB injury [35].

### Integration of Biomechanical Testing and Simulation with Imaging for Enhanced SCB Analysis

Given that the primary aetiology involves mechanical loading, integrating CT imaging with assessments of mechanical strain in SCB is essential. For example, it has been postulated that two-year-old Thoroughbred racehorses experience high strain in the diaphysis and metaphysis of metacarpal/tarsal when training commences due to lower BVF and increased trabecular space [47]. However, there is little evidence on the actual strains within the bone in these cases. A recent study integrated mechanical testing, digital image correlation (a surface strain measurement technique) and standing CT imaging to investigate SCB fatigue injury in the third metacarpal bones of Thoroughbred racehorses. The study revealed increased strain under experimental loading in the parasagittal grooves of bones with SCB lesions, as detected by CT, compared to those without lesions [48].

In human medicine, the application of computational finite element (FE) models based on CT imaging has revolutionized the prediction of hip and vertebral fractures [49, 50, 51]. A similar approach could enhance the prediction of SCB failure and stress fractures in horses. Early work by Harrison et al. [52] developed a subject-specific FE model of the equine metacarpophalangeal joint that successfully incorporated deformable cartilage and elastic ligaments, but modelled bone as rigid. The model was validated against experimental data, demonstrating the importance of subject-specific geometry, in accurately predicting joint mechanics. Frazer et al. (2017, 2019) developed a CT-based FE model of the equine stifle joint [53, 54]. Using the same model with the same bone density distribution, they explored the effects of SCB cysts, with idealised geometries, on local bone stresses. Their studies revealed that the presence of SBCs or voids increased local stress levels, potentially contributing to further bone damage and persistence of these lesions. It is important to recognize that fatigue damage, including stress fractures or other lesions involve complex interactions influenced by bone repair and adaptation at the microstructural level. A combined strategy incorporating longitudinal CT scans, biomechanical testing, and computational modelling incorporating adaptation and repair components could facilitate more accurate predictions of injury and deepen our understanding of disease initiation and progression. One critical factor in developing such image-based pipelines that can accurately relate macroscale joint behaviour to its intrinsic microstructure and function is the precise evaluation and examination of SCB structure-functional relationships, which can then inform FE models of the joint. A recent study by Moshage et al. (2022) employed a comprehensive approach to establish the relationship between CT images, micro-CT data, and the elastic modulus of bone cores from juvenile horses [55]. This work highlights the importance of considering age-specific density-modulus relationships when developing FE models, ultimately leading to more accurate predictions of mechanical behaviour in equine bones. Micro-CT offers high resolution, submicron, three-dimensional images enabling the visualization of bone microarchitecture with exquisite detail.

### SCB Structure–function Relationships: The Role of Micro-CT

When scanning SCB, the high resolution of micro-CT allows for detailed visualization of the porous structure of the SCB plate, the microstructure and surface shape of the tidemark, and the trabecular structure of SCB [2, 5, 16, 56]. Recent studies have shown that the superficial SCB within the first 10 mm, which is most susceptible to injury in racehorses, is less stiff than the deeper SCB. This difference persists under various loading conditions, including single impact and cyclic loading [2, 5, 57]. Additionally, superficial SCB microdamage, most commonly observed in the equine fetlock joint of racehorses, has been found to have the greatest influence on the bone’s mechanical response. While visualizing microcracks at the tidemark is usually a limitation, most micro-CT scanners can image microdamage and microfractures wider than a few micrometres or those containing highly mineralized material [5, 58].

One way to tackle the limitation of micro-CT in measuring fine microcracks has been the use of contrast-enhanced CT with lead-uranyl acetate. This heavy metal label accumulates in microdamage areas, enhancing electron density and improving visualization in micro-CT. Recent studies have developed protocols for quantifying such microdamage in SCB [2, 59]. Luedke et al. (2022) utilized contrast-enhanced micro-CT alongside mechanical testing to examine the interplay between fracture toughness and microdamage in the proximal sesamoid bones (PSBs) of racehorses [60]. They observed microdamage in the SCB independent of fracture events, although their study focused on the palmar aspect of the sesamoids where microdamage was minimal.

The loading conditions that SCB in racehorses experience is high-rate and repetitive. Understanding how this type of dynamic loading influences the microarchitecture and biomechanical responses of the SCB is crucial. One of the challenges in accurately measuring the response of SCB to mechanical loading is that SCB is integrated with its adjacent cartilage, complicating isolated testing without disrupting the most superficial layer of the SCB and the calcified cartilage, where most in vivo microdamage occurs. To address this challenge, micro-CT imaging and mechanical testing have been combined with digital image correlation (DIC). This approach allows testing of the cartilage-bone unit as a whole while extracting their individual mechanical properties [61]. Using a similar approach, Malekipour et al. (2022) demonstrated that microdamage induced in the SCB of racehorses by repetitive high rate loading in vivo reduced its mechanical stiffness under a single impact load, leading to considerably higher localized strains in damaged areas [5]. Pearce et al. (2022) utilized micro-CT alongside mechanical testing and DIC to investigate correlations between microstructural attributes to the impact response of SCB in Thoroughbred racehorses from various sites within the MC3 joint [2]. They found that bone density and mechanical properties varied across different joint locations. Particularly, in regions subjected to higher stress, i.e. the palmar aspect of the metacarpal condyles and proximal sesamoid SCB, exhibited higher stiffness and adaptive bone structure [2]. Shaktivesh et al. (2024) exclusively tested SCB and found high localized strains within SCB in relation to microdamage, under a spectrum of high-rate cyclic compression, similar to that applied to the bone in vivo [16]. Additionally, their results suggest that structural and mechanical properties of SCB exhibit variability throughout its depth from the joint surface [2, 5, 62].

While these methods provide valuable insights into the overall behaviour of the tissue, information about the internal strains and stresses within the SCB has been limited. However, with the increased use of micro-CT imaging and advancements in computational power, a combined approach integrating finite element (FE) modelling, micro-CT, and experimental data holds promise for identifying predictive parameters to predict fatigue injury in SCB, such as the volume of bone under high stresses [63]. Additionally, it is important to acknowledge that micro-CT may have limitations in accurately predicting SCB mechanical response [64], potentially due to other influencing factors such as material heterogeneity, anisotropy, and the complex geometry of bone. Therefore, a comprehensive understanding of SCB behaviour requires an integrative approach that combines high-resolution imaging, robust computational models, and experimental validation to improve the predictive accuracy for fatigue injuries and inform better clinical decisions.

### Future Directions and Use of AI in SCB Imaging Using CT and Micro-CT

Recently, the integration of Artificial intelligence (AI) and machine learning (ML) with human medical imaging has helped overcome some of the limitations of traditional methodologies in fracture detection, classification [65, 66, 67] as well as for segmentation procedures required for developing FE models. Manual segmentation, which is both time-consuming and prone to error, along with the subjective nature of diagnostic methods, are among the limitations of current methods [68]. Additionally, SCB biomechanics is a complex research area which requires analysing the interactions between mechanical loads and the responses of heterogenous and nonlinear biological tissues, especially in fatigue injury and stress fracture research. While AI, especially deep learning methods, holds promise in this area [69], these advances are in the initial stages of application within equine and other large animal models [70, 71]. AI has been used in canine models to identify fracture in long bones using radiography [72]. The slower progression in this area, compared to AI’s application in human studies, can be attributed to the smaller datasets available for these animals as compared to human datasets, which poses significant challenges for developing effective AI models [71]. Nonetheless, the adoption of standing CT in the equine domain is contributing to the creation of larger, more detailed datasets, which may improve AI application in veterinary settings. Additionally, recent improvements in algorithms, particularly those that employ data augmentation, are enhancing AI capability to process medical images of equine and large animals [65]. Rytky et al. (2021) applied deep learning segmentation to micro-CT images for assessing the three-dimensional morphology of calcified cartilage (CC) in rabbits [73]. They found strong correlations with traditional histological methods, with Dice scores of 0.891 for histology and 0.807 for micro-CT segmentation. It provided detailed insights into CC thickness variations across different anatomical regions, suggesting micro-CT as a superior method for analysing dynamic changes in cartilage mineralization. These advancements aid in predicting and diagnosing abnormalities and perform complex tasks such as segmenting different bone structures for FE models [74]. More importantly, techniques like transfer learning and data synthesis are further broadening AI applications, enabling the adaptation of models originally trained on specific datasets to new tasks, providing potential application of AI in both diagnostic accuracy and operational efficiency. For example, Amodeo et al. (2021) developed a maxillofacial fracture detection system using convolutional neural networks pre-trained on non-medical images, which was then re-trained and fine-tuned using CT scans to classify future CTs as either “fracture” or “no Fracture” [75]. The system achieved an 80% accuracy in classifying fractures, categorizing patients as fractured if two consecutive slices had a fracture probability higher than 0.99. This approach demonstrated the potential to assist radiologists by reducing diagnostic errors and delays, minimizing the risk of human error, and decreasing unnecessary hospitalizations. These studies highlight the potential of integrating advanced imaging with deep learning to improve diagnostic accuracy and clinical outcomes. Additionally, ethical considerations and the need for standardization in AI applications within veterinary medicine need to be investigated.

## Conclusion

In conclusion, the integration of advanced imaging technologies, such as CT and micro-CT, has dramatically enhanced our understanding of SCB biomechanics, particularly in large animal models like horses. These technologies have facilitated detailed, non-destructive examination of SCB microarchitecture, revealing critical insights into its role in joint integrity and disease progression, such as OA, SCB injury and stress fractures. The advent of standing CT machines, along with FBCT and CBCT technologies, has made SCB imaging more practical and accessible, enabling longitudinal studies and detailed diagnoses in live, standing animals. Despite these advances, challenges remain in predicting and detecting SCB pathology early. Integrating mechanical testing, digital image correlation, and finite element modelling shows promise in addressing these challenges by correlating imaging data with mechanical and structural analyses to better predict SCB failure. Furthermore, the emerging field of AI and ML in medical imaging holds significant potential for improving diagnostic accuracy and operational efficiency, despite being relatively novel in veterinary medicine. By continuing to refine these technologies and adopting a multidisciplinary approach, we can advance our understanding of SCB biomechanics and enhance clinical management of joint disease in both animals and humans.

## Data Availability

No datasets were generated or analysed during the current study.

## References

[1] Duncan H, Jundt J, Riddle JM, Pitchford W, Christopherson T. The tibial subchondral plate. J Bone Jt Surg. 1987;69:1212–20. 3667650

[2] Pearce DJ, Hitchens PL, Malekipour F, Ayodele B, Lee PVS, Whitton RC. Biomechanical and Microstructural Properties of Subchondral Bone From Three Metacarpophalangeal Joint Sites in Thoroughbred Racehorses. Front Vet Sci 2022;9. 10.3389/fvets.2022.923356. 10.3389/fvets.2022.923356 PMC9277662 35847629

[3] Findlay DM, Kuliwaba JS. Bone-cartilage crosstalk: A conversation for understanding osteoarthritis. Bone Res 2016;4. 10.1038/boneres.2016.28. 10.1038/boneres.2016.28 PMC5028726 27672480

[4] Boyde A, Haroon Y, Jones SJ, Riggs CM. Three dimensional structure of the distal condyles of the third metacarpal bone of the horse. Equine Vet J. 1999;31:122–9. 10.1111/j.2042-3306.1999.tb03805.x. 10213424 10.1111/j.2042-3306.1999.tb03805.x

[5] Malekipour F, Hitchens PL, Whitton RC, Vee-Sin Lee P. Effects of in vivo fatigue-induced microdamage on local subchondral bone strains. J Mech Behav Biomed Mater 2022;136:105491. 10.1016/j.jmbbm.2022.105491. This study is important because it investigates the effects of *in vivo* fatigue-induced microdamage on local subchondral bone strains, highlighting the relationship between mechanical properties and microdamage, which is crucial for understanding SCB biomechanics and its role in joint diseases. 10.1016/j.jmbbm.2022.105491 36198232

[6] Malekipour F, Whitton CR, Lee PVS. Stiffness and energy dissipation across the superficial and deeper third metacarpal subchondral bone in Thoroughbred racehorses under high-rate compression. J Mech Behav Biomed Mater. 2018;85:51–6. 10.1016/j.jmbbm.2018.05.031. 29852352 10.1016/j.jmbbm.2018.05.031

[7] Malekipour F. Chris Whitton LPVS. Spatial distribution of strain in equine distal metacarpal subchondral bone: a microCT-based finite element model. Aust. New Zeal. Orthop. Res. Soc. Canberra, Aust., 2019.

[8] Martig S, Hitchens PL, Stevenson MA, Whitton1 RC. Subchondral bone morphology in the metacarpus of racehorses in training changes with distance from the articular surface but not with age. J Anat 2018;232:919–30. 10.1177/0972063414539595. 10.1111/joa.12794 PMC5979622 29446086

[9] Burr D. Anatomy and physiology of the mineralized tissues: Role in the pathogenesis of osteoarthrosis. Osteoarthr Cartil. 2004;12:20–30. 10.1016/j.joca.2003.09.016. 10.1016/j.joca.2003.09.016 14698637

[10] Riggs CM, Whitehouse GH, Boyde A. Structural variation of the distal condyles of the third metacarpal and third metatarsal bones in the horse. Equine Vet J. 1999;31:130–9. 10.1111/j.2042-3306.1999.tb03806.x. 10213425 10.1111/j.2042-3306.1999.tb03806.x

[11] Stewart HL, Kawcak CE. The Importance of Subchondral Bone in the Pathophysiology of Osteoarthritis. Front Vet Sci. 2018;5:1–9. 10.3389/fvets.2018.00178. 30211173 10.3389/fvets.2018.00178 PMC6122109

[12] Goldring SR, Goldring MB. Changes in the osteochondral unit during osteoarthritis: Structure, function and cartilage bone crosstalk. Nat Rev Rheumatol. 2016;12:632–44. 10.1038/nrrheum.2016.148. 27652499 10.1038/nrrheum.2016.148

[13] Whitton RC, Mirams M, Mackie EJ, Anderson G a, Seeman E. Exercise-induced inhibition of remodelling is focally offset with fatigue fracture in racehorses. Osteoporos Int 2013;24:2043–8. 10.1007/s00198-013-2291-z. 10.1007/s00198-013-2291-z 23371360

[14] Eckstein F, Müller-Gerbl M, Putz R. Distribution of subchondral bone density and cartilage thickness in the human patella. J Anat 1992;180 ( Pt 3:425–33. PMC1259644 1487436

[15] Riggs CM, Whitehouse GH, Boyde A. Structural variation of the distal condyles of the third metacarpal and the third metatarsal bones in the horse. Equine Vet J. 1999;31:140–8. 10.1111/j.2042-3306.1999.tb03807.x. 10213425 10.1111/j.2042-3306.1999.tb03806.x

[16] Shaktivesh S, Malekipour F, Whitton RC, Vs P. Journal of the Mechanical Behavior of Biomedical Materials Mechanical response of local regions of subchondral bone under physiological loading conditions. J Mech Behav Biomed Mater. 2024;152: 106405. 10.1016/j.jmbbm.2024.106405. 38271752 10.1016/j.jmbbm.2024.106405

[17] Oláh T, Cai X, Michaelis JC, Madry H. Comparative anatomy and morphology of the knee in translational models for articular cartilage disorders. Part I: Large animals Ann Anat. 2021;235: 151680. 10.1016/j.aanat.2021.151680. 33548412 10.1016/j.aanat.2021.151680

[18] Kuyinu EL, Narayanan G, Nair LS, Laurencin CT. Animal models of osteoarthritis: Classification, update, and measurement of outcomes. J Orthop Surg Res. 2016;11:1–27. 10.1186/s13018-016-0346-5. 26837951 10.1186/s13018-016-0346-5 PMC4738796

[19] McCoy AM. Animal Models of Osteoarthritis: Comparisons and Key Considerations. Vet Pathol. 2015;52:803–18. 10.1177/0300985815588611. 26063173 10.1177/0300985815588611

[20] Gregory MH, Capito N, Kuroki K, Stoker AM, Cook JL, Sherman SL. A Review of Translational Animal Models for Knee Osteoarthritis. Arthritis. 2012;2012:1–14. 10.1155/2012/764621. 10.1155/2012/764621 PMC3541554 23326663

[21] Oláh T, Cucchiarini M, Madry H. Subchondral bone remodeling patterns in larger animal models of meniscal injuries inducing knee osteoarthritis – a systematic review. Knee Surgery, Sport Traumatol Arthrosc. 2023;31:5346–64. 10.1007/s00167-023-07579-6. 10.1007/s00167-023-07579-6 PMC10719152 37742232

[22] Boyde A. The Bone Cartilage Interface and Osteoarthritis. Calcif Tissue Int. 2021;109:303–28. 10.1007/s00223-021-00866-9. 34086084 10.1007/s00223-021-00866-9 PMC8403126

[23] Posukonis MN, Daglish J, Wright IM, Kawcak CE. Novel computed tomographic analysis demonstrates differences in patterns of bone mineral content between fracture configurations in distal condylar fractures of the third metacarpal/metatarsal bones in 97 Thoroughbred racehorses. Am J Vet Res. 2022;83:1–9. 10.2460/ajvr.22.03.0060. 10.2460/ajvr.22.03.0060 36327168

[24] Turlo AJ, McDermott BT, Barr ED, Riggs CM, Boyde A, Pinchbeck GL, et al. Gene expression analysis of subchondral bone, cartilage, and synovium in naturally occurring equine palmar/plantar osteochondral disease. J Orthop Res. 2022;40:595–603. 10.1002/jor.25075. 33993513 10.1002/jor.25075

[25] McIlwraith CW, Frisbie DD, Kawcak CE. The horse as a model of naturally occurring osteoarthritis. Bone Joint Res. 2012;1:297–309. 10.1302/2046-3758.111.2000132. 23610661 10.1302/2046-3758.111.2000132 PMC3626203

[26] Frisbie D, Cross M, McIlwraith C. A comparative study of articular cartilage thickness in the stifle of animal species used in human pre-clinical studies compared to articular cartilage thickness in the human knee. Vet Comp Orthop Traumatol 2006;19:142–6. 16971996

[27] Muir P, Peterson AL, Sample SJ, Scollay MC, Markel MD, Kalscheur VL. Exercise-induced metacarpophalangeal joint adaptation in the Thoroughbred racehorse. J Anat. 2008;213:706–17. 10.1111/j.1469-7580.2008.00996.x. 19094186 10.1111/j.1469-7580.2008.00996.x PMC2666139

[28] Barr ED, Pinchbeck GL, Clegg PD, Boyde a, Riggs CM. Post mortem evaluation of palmar osteochondral disease (traumatic osteochondrosis) of the metacarpo/metatarsophalangeal joint in Thoroughbred racehorses. Equine Vet J 2009;41:366–71. 10.2746/042516409X368372. 10.2746/042516409x368372 19562898

[29] Matcuk GR, Mahanty SR, Skalski MR, Patel DB, White EA, Gottsegen CJ. Stress fractures: pathophysiology, clinical presentation, imaging features, and treatment options. Emerg Radiol 2016:1–11. 10.1007/s10140-016-1390-5. 10.1007/s10140-016-1390-5 27002328

[30] Ibad HA, De Cesar NC, Shakoor D, Sisniega A, Liu SZ, Siewerdsen JH, et al. Computed Tomography: State-of-the-Art Advancements in Musculoskeletal Imaging. Invest Radiol. 2023;58:99–110. 10.1097/RLI.0000000000000908. 35976763 10.1097/RLI.0000000000000908 PMC9742155

[31] Kasaeian A, Roemer FW, Ghotbi E, Ibad HA, He J, Wan M, et al. Subchondral bone in knee osteoarthritis: bystander or treatment target? Skeletal Radiol. 2023;52:2069–83. 10.1007/s00256-023-04422-4. 37646795 10.1007/s00256-023-04422-4 PMC12224767

[32] Steel C, Ahern B, Zedler S, Vallance S, Galuppo L, Richardson J, et al. Comparison of Radiography and Computed Tomography for Evaluation of Third Carpal Bone Fractures in Horses. Animals 2023;13:0–15. 10.3390/ani13091459. 10.3390/ani13091459 PMC10177357 37174496

[33] Ammann L, Ohlerth S, Fürst AE, Jackson MA. Differences of morphological attributes between 62 proximal and distal subchondral cystic lesions of the proximal phalanx as determined by radiography and computed tomography. Am J Vet Res. 2022;83:1–9. 10.2460/ajvr.22.04.0071. 10.2460/ajvr.22.04.0071 36315450

[34] Nagy A, Boros K, Dyson S. Magnetic Resonance Imaging, Computed Tomographic and Radiographic Findings in the Metacarpophalangeal Joints of 40 Non-Lame Thoroughbred Yearlings. Animals 2023;13. 10.3390/ani13223466. 10.3390/ani13223466 PMC10668665 38003084

[35] Wright I, Minshall G, Young N, Riggs C. Fractures in Thoroughbred racing and the potential for pre-race identification of horses at risk. Equine Vet J 2024:424–36. 10.1111/evj.14046. 10.1111/evj.14046 38200406

[36] Cianci JM, Wulster KB, Richardson DW, Stefanovski D, Ortved KF. Computed tomographic assessment of fracture characteristics and subchondral bone injury in Thoroughbred racehorses with lateral condylar fractures and their relationship to outcome. Vet Surg. 2022;51:426–37. 10.1111/vsu.13770. 35165910 10.1111/vsu.13770

[37] Williamson AJ, Sims NA, Thomas CDL, Lee PVS, Stevenson MA, Whitton RC. Biomechanical testing of the calcified metacarpal articular surface and its association with subchondral bone microstructure in Thoroughbred racehorses. Equine Vet J. 2018;50:255–60. 10.1111/evj.12748. 28833497 10.1111/evj.12748

[38] Dubois MS, Morello S, Rayment K, Markel MD, Vanderby R, Kalscheur VL, et al. Computed tomographic imaging of subchondral fatigue cracks in the distal end of the third metacarpal bone in the thoroughbred racehorse can predict crack micromotion in an ex-vivo model. PLoS One 2014;9. 10.1371/journal.pone.0101230. 10.1371/journal.pone.0101230 PMC4117462 25077477

[39] Brounts SH, Henry T, Lund JR, Chris RW, Ergun DL, Muir P. Use of a novel helical fan beam imaging system for computed tomography of the distal limb in sedated standing horses: 167 cases (2019–2020). J Am Vet Med Assoc. 2022;260:1351–60. 10.2460/javma.21.10.0471. 35943949 10.2460/javma.21.10.0439

[40] Brounts SH, Henry T, Lund JR, Chris RW, Ergun DL, Muir P. Use of a novel helical fan beam imaging system for computed tomography of the head and neck in sedated standing horses: 120 cases (2019–2020). J Am Vet Med Assoc. 2022;260:1361–8. 10.2460/javma.21.10.0471. 35943950 10.2460/javma.21.10.0471

[41] Mathee N, Robert M, Higgerty SM, Fosgate GT, Rogers AL, d’Ablon X, et al. Computed tomographic evaluation of the distal limb in the standing sedated horse: Technique, imaging diagnoses, feasibility, and artifacts. Vet Radiol Ultrasound. 2023;64:243–52. 10.1111/vru.13182. 36373276 10.1111/vru.13182

[42] Lin ST, Bolas NM, Sargan DR, Restif O, Peter VG, Pokora R, et al. Comparison of cone-beam and fan-beam computed tomography and low-field magnetic resonance imaging for detection of proximal phalanx dorsoproximal osteochondral defects. Equine Vet J 2023:484–93. 10.1111/evj.13973. 10.1111/evj.13973 37488678

[43] Stewart HL, Siewerdsen JH, Selberg KT, Bills KW, Kawcak CE. Cone-beam computed tomography produces images of numerically comparable diagnostic quality for bone and inferior quality for soft tissues compared with fan-beam computed tomography in cadaveric equine metacarpophalangeal joints. Vet Radiol Ultrasound. 2023;64:1033–6. 10.1111/vru.13309. 37947254 10.1111/vru.13309

[44] McKay RM, Vapniarsky N, Hatcher D, Carr N, Chen S, Verstraete FJM, et al. The Diagnostic Yield of Cone-Beam Computed Tomography for Degenerative Changes of the Temporomandibular Joint in Dogs. Front Vet Sci. 2021;8:1–14. 10.3389/fvets.2021.720641. 10.3389/fvets.2021.720641 PMC8371634 34422949

[45] Lin ST, Foote AK, Bolas NM, Peter VG, Pokora R, Patrick H, et al. Three-Dimensional Imaging and Histopathological Features of Third Metacarpal/Tarsal Parasagittal Groove and Proximal Phalanx Sagittal Groove Fissures in Thoroughbred Horses. Animals 2023;13. 10.3390/ani13182912. 10.3390/ani13182912 PMC10525482 37760312

[46] Ciamillo SA, Wulster KB, Gassert TM, Richardson DW, Brown KA, Stefanovski D, et al. Prospective, longitudinal assessment of subchondral bone morphology and pathology using standing, cone-beam computed tomography in fetlock joints of 2-year-old Thoroughbred racehorses in their first year of training. Equine Vet J 2024:1–14. 10.1111/evj.14048. 10.1111/evj.14048 38247205

[47] Boyde A, Firth EC. Musculoskeletal responses of 2-year-old Thoroughbred horses to early training. 8. Quantitative back-scattered electron scanning electron microscopy and confocal fluorescence microscopy of the epiphysis of the third metacarpal bone. N Z Vet J 2005;53:123–32. 10.1080/00480169.2005.36489. 10.1080/00480169.2005.36489 15846396

[48] Irandoust S, Whitton RC, Muir P, Henak CR. Subchondral Bone Fatigue Injury in the Parasagittal Condylar Grooves of the Distal End of the Third Metacarpal Bone in Thoroughbred Racehorses Elevates Site-Specific Strain Concentration. Ssrn. 2023;155: 106561. 10.1016/j.jmbbm.2024.106561. 10.1016/j.jmbbm.2024.106561 38678748

[49] Silva MJ, Keaveny TM, Hayes WC. Computed tomography-based finite element analysis predicts failure loads and fracture patterns for vertebral sections. J Orthop Res. 1998;16:300–8. 10.1002/jor.1100160305. 9671924 10.1002/jor.1100160305

[50] Keaveny TM, Clarke BL, Cosman F, Orwoll ES, Siris ES, Khosla S, et al. Biomechanical Computed Tomography analysis (BCT) for clinical assessment of osteoporosis. Osteoporos Int. 2020;31:1025–48. 10.1007/s00198-020-05384-2. 32335687 10.1007/s00198-020-05384-2 PMC7237403

[51] Viceconti M, Qasim M, Bhattacharya P, Li X. Are CT-Based Finite Element Model Predictions of Femoral Bone Strengthening Clinically Useful? Curr Osteoporos Rep. 2018;16:216–23. 10.1007/s11914-018-0438-8. 29656377 10.1007/s11914-018-0438-8 PMC5945796

[52] Harrison SM, Whitton RC, Kawcak CE, Stover SM, Pandy MG. Evaluation of a subject-specific finite-element model of the equine metacarpophalangeal joint under physiological load. J Biomech. 2014;47:65–73. 10.1016/j.jbiomech.2013.10.001. 24210848 10.1016/j.jbiomech.2013.10.001

[53] Frazer LL, Santschi EM, Fischer KJ. Impact of a void in the equine medial femoral condyle on bone stresses and peak contact pressures in a finite element model. Vet Surg. 2019;48:237–46. 10.1111/vsu.13139. 30556152 10.1111/vsu.13139

[54] Frazer LL, Santschi EM, Fischer KJ. The impact of subchondral bone cysts on local bone stresses in the medial femoral condyle of the equine stifle joint. Med Eng Phys. 2017;48:158–67. 10.1016/j.medengphy.2017.06.019. 28690042 10.1016/j.medengphy.2017.06.019

[55] Moshage SG, McCoy AM, Kersh ME. Elastic Modulus and Its Relation to Apparent Mineral Density in Juvenile Equine Bones of the Lower Limb. J Biomech Eng 2023;145. 10.1115/1.4062488. 10.1115/1.4062488 37144881

[56] Marsiglia MF, Yamada ALM, Agreste FR, de Sá LRM, Nieman RT, da Silva LCLC. Morphological analysis of third metacarpus cartilage and subchondral bone in Thoroughbred racehorses: An ex vivo study. Anat Rec. 2022;305:3385–97. 10.1002/ar.24918. 10.1002/ar.24918 35338614

[57] Martig S, Hitchens PL, Lee PVS, Whitton RC. The relationship between microstructure, sti ff ness and compressive fatigue life of equine subchondral bone. J Mech Behav Biomed Mater. 2020;101: 103439. 10.1016/j.jmbbm.2019.103439. 31557658 10.1016/j.jmbbm.2019.103439

[58] Whitton RC, Ayodele BA, Hitchens PL, Mackie EJ. Subchondral bone microdamage accumulation in distal metacarpus of Thoroughbred racehorses. Equine Vet J. 2018;50:766–73. 10.1111/evj.12948. 29660153 10.1111/evj.12948

[59] Ayodele BA, Malekipour F, Pagel CN, Mackie EJ, Whitton RC. Assessment of subchondral bone microdamage quantification using contrast‐enhanced imaging techniques. J Anat 2024:1–12. 10.1111/joa.14035. 10.1111/joa.14035 PMC11161821 38481117

[60] Luedke LK, Ilevbare P, Noordwijk KJ, Palomino PM, McDonough SP, Palmer SE, et al. Proximal sesamoid bone microdamage is localized to articular subchondral regions in Thoroughbred racehorses, with similar fracture toughness between fracture and controls. Vet Surg. 2022;51:952–62. 10.1111/vsu.13816. 35672916 10.1111/vsu.13816 PMC13378172

[61] Malekipour F, Whitton C, Oetomo D, Lee PVS. Shock absorbing ability of articular cartilage and subchondral bone under impact compression. J Mech Behav Biomed Mater. 2013;26:127–35. 10.1016/j.jmbbm.2013.05.005. 23746699 10.1016/j.jmbbm.2013.05.005

[62] Shaktivesh S, Malekipour F, Whitton RC, Vs P, Whitton C, Lee PV. Mechanical response of local regions of subchondral bone under physiological loading conditions. J Mech Behav Biomed Mater. 2024;152: 106405. 10.1016/j.jmbbm.2024.106405. 38271752 10.1016/j.jmbbm.2024.106405

[63] Koshyk A, Pohl AJ, Takahashi Y, Scott WM, Sparks HD, Edwards WB. Influence of microarchitecture on stressed volume and mechanical fatigue behaviour of equine subchondral bone. Bone 2024;182:117054. 10.1016/j.bone.2024.117054. This study is important because it examines how the microarchitecture of equine subchondral bone influences its stressed volume and mechanical fatigue behavior. A combined approach integrating FE modelling, micro-CT, and experimental data holds promise for identifying predictive parameters to predict fatigue injury in SCB, such as the volume of bone under high stresses. 10.1016/j.bone.2024.117054 38395248

[64] Malekipour F, Whitton RC, Lee PV-S. Distribution of mechanical strain in equine distal metacarpal subchondral bone: A microCT-based finite element model. Med Nov Technol Devices 2020;6:100036. 10.1016/j.medntd.2020.100036.

[65] Kim T, Goh TS, Lee JS, Lee JH, Kim H, Jung ID. Transfer learning-based ensemble convolutional neural network for accelerated diagnosis of foot fractures. Phys Eng Sci Med. 2023;46:265–77. 10.1007/s13246-023-01215-w. 36625995 10.1007/s13246-023-01215-w

[66] Ataei A, Eggermont F, Verdonschot N, Lessmann N, Tanck E. The effect of deep learning-based lesion segmentation on failure load calculations of metastatic femurs using finite element analysis. Bone. 2024;179: 116987. 10.1016/j.bone.2023.116987. 38061504 10.1016/j.bone.2023.116987

[67] Dankelman LHM, Schilstra S, IJpma FFA, Doornberg JN, Colaris JW, Verhofstad MHJ, et al. Artificial intelligence fracture recognition on computed tomography: review of literature and recommendations. Eur J Trauma Emerg Surg. 2023;49:681–91. 10.1007/s00068-022-02128-1. 36284017 10.1007/s00068-022-02128-1 PMC10175338

[68] Burti S, Banzato T, Coghlan S, Wodzinski M, Bendazzoli M, Zotti A. Artificial intelligence in veterinary diagnostic imaging: Perspectives and limitations. Res Vet Sci. 2024;175: 105317. 10.1016/j.rvsc.2024.105317. 38843690 10.1016/j.rvsc.2024.105317

[69] Yang Z, Yu CH, Buehler MJ. Deep learning model to predict complex stress and strain fields in hierarchical composites. Sci Adv. 2021;7:1–10. 10.1126/SCIADV.ABD7416. 10.1126/sciadv.abd7416 PMC8034856 33837076

[70] Pereira AI, Franco-Gonçalo P, Leite P, Ribeiro A, Alves-Pimenta MS, Colaço B, et al. Artificial Intelligence in Veterinary Imaging: An Overview. Vet Sci 2023;10. 10.3390/vetsci10050320. 10.3390/vetsci10050320 PMC10223052 37235403

[71] Hennessey E, DiFazio M, Hennessey R, Cassel N. Artificial intelligence in veterinary diagnostic imaging: A literature review. Vet Radiol Ultrasound. 2022;63:851–70. 10.1111/vru.13163. 36468206 10.1111/vru.13163

[72] Ergun GB, Guney S. Classification of Canine Maturity and Bone Fracture Time Based on X-Ray Images of Long Bones. IEEE Access. 2021;9:109004–11. 10.1109/ACCESS.2021.3101040.

[73] Rytky SJO, Huang L, Tanska P, Tiulpin A, Panfilov E, Herzog W, et al. Automated analysis of rabbit knee calcified cartilage morphology using micro-computed tomography and deep learning. J Anat. 2021;239:251–63. 10.1111/joa.13435. 33782948 10.1111/joa.13435 PMC8273618

[74] Hespel AM, Zhang Y, Basran PS. Artificial intelligence 101 for veterinary diagnostic imaging. Vet Radiol Ultrasound. 2022;63:817–27. 10.1111/vru.13160. 36514230 10.1111/vru.13160

[75] Amodeo M, Abbate V, Arpaia P, Cuocolo R, Orabona GD, Murero M, et al. Transfer learning for an automated detection system of fractures in patients with maxillofacial trauma. Appl Sci 2021;11. 10.3390/app11146293.

